# Prevalence of self-reported stomach symptoms after consuming milk among indigenous Sami and non-Sami in Northern- and Mid-Norway – the SAMINOR study

**DOI:** 10.3402/ijch.v74.25762

**Published:** 2015-02-17

**Authors:** Ketil Lenert Hansen, Magritt Brustad, Knut Johnsen

**Affiliations:** 1 Centre for Sami Health Research, Institute of Community Medicine, UiT The Arctic University of Norway, Tromsø, Norway; 2 Institute of Community Medicine, UiT The Arctic University of Norway, Tromsø, Norway; 3 The Finnmark Clinic, University Hospital of Northern Norway, Karasjok, Norway

**Keywords:** Sami, epidemiology, ethnicity, health, milk intolerance, lactose intolerance, hypolactasia, Arctic

## Abstract

**Objective:**

The main purpose of this work was to identify the prevalence of self-reported stomach symptoms after consuming milk among Sami and non-Sami adults.

**Study design:**

A cross-sectional population-based study (the SAMINOR study). Data were collected by self-administrated questionnaires.

**Method:**

SAMINOR is a population-based study of health and living conditions conducted in 24 municipalities in Northern Norway during 2003 and 2004. The present study included 15,546 individuals aged between 36 and 79, whose ethnicity was categorized as Sami (33.4%), Kven (7.3%) and Norwegian majority population (57.2%).

**Results:**

Sami respondents had a higher prevalence of self-reported stomach symptoms after consuming milk than the Norwegian majority population. The reporting was highest among Sami females (27.1%). Consumption of milk and dairy products (yoghurt and cheese) was high among all the ethnic groups. However, significantly more Sami than non-Sami never (or rarely) consume milk or cheese, and individuals who reported stomach symptoms after consuming milk had an significant lower intake of dairy products than those not reporting stomach symptoms after consuming dairy products. Sami reported general abdominal pain more often than the majority population. The adjusted models show a significant effect of Sami ethnicity in both men and women on self-reported stomach symptoms after consuming milk. In females, the odds ratio (OR)=1.77 (p=0.001) and in males OR=1.64 (p=0.001).

**Conclusion:**

Our study shows that the Sami population reported more stomach symptoms after consuming milk, suggesting a higher prevalence of milk intolerance among the Sami population than the Norwegian majority population.

Milk is an important everyday source of nutrition in Northern Europe (1). In Northern Europe, lactase persistence is common, which allows the majority of the population to consume milk and dairy products (2), while approximately 70% of the total adult human population world-wide has hypolactasia (3, 4). In adult mammals, lactose tolerance normally disappears after weaning (3). The prevalence of adult lactose tolerance varies between different ethnic groups and populations. In populations where the frequency of lactase persistence genotype is rather low, one would expect elevated self-reported stomach symptoms after consumption of milk (5, 6).

In the majority of subjects with lactose intolerance, the clinical symptoms will occur before 12 years of age, but this is dependent on the amount of intake of lactose. The most common gastrointestinal symptoms that characterize intolerance to lactose are abdominal pain in the stomach, diarrhoea, bloating and flatulence (7). However, current studies have shown that subjects with intolerance to lactose tend to reduce their consumption of milk and dairy products, which is unsurprising since they suffer from symptoms after milk consumption (8).

Northern Europeans, North Americans and Australasians have the highest prevalence rates of lactose tolerance; ranging from 74% to more than 90% (3, 9), while it is very common to be lactose intolerant in South American, African and Asian populations, where 50% or more of the population has lactose non-persistence, and in some Asian countries the figure is almost 100% (4, 10). The population-based prevalence of lactose intolerance is very rare (2–8%) in Swedish, Danish and Norwegian majority populations (4).

Among indigenous populations in North America, Siberia, Greenland and Oceania, the prevalence of milk intolerance is more than 60% and in some tribes even close to 100% (6, 10, 11). In Finland, which may have the most reliable and valid estimates of the prevalence of lactose intolerance, it has been documented at 17% among the Finnish-speaking Finns, while the lowest prevalence (8%) has been found on the south and west coasts in the Swedish-speaking populations of Finns and Swedes. In northernmost Finland, the prevalence varies from 25 to 60% among the Finnish Sami population (10). Similar studies have been conducted among Sami people in Kola, Russia, which also show a high prevalence of lactose intolerance, as in other indigenous circumpolar populations of Eurasia (12, 13). Remarkably, in subjects of mixed ethnicity, a lower prevalence is detected in the native ethnic groups (4).

The Sami are the indigenous people of *Sápmi*, a territory comprising parts of Arctic Norway, Sweden, Finland, and Russia's Kola Peninsula (14). The Sami have traditionally been engaged in a variety of livelihoods, including farming, fishing, trapping and reindeer husbandry (breeding and herding). Traditional means of subsistence – continuing to this day – such as reindeer husbandry, often in combination with small-scale fishing and agriculture, form the economic backbone of Sami communities (15). The “traditional Sami diet” has been characterized by high intake of fatty fish, red meat (primarily reindeer), fat, blood and organs, wild berries and boiled, unfiltered coffee, and low intakes of cultivated vegetables and fruit, bread and fibre (16). However, today the Norwegian Sami have a diverse dietary pattern (17). The question of whether the Sami used milk as an additional source of proteins, lipids and sugar (lactose) is of particular interest (12). Ethnographic literature shows that the reindeer dairy farming of the Scandinavian Sami developed rather late (in the 18th century) and did not spread across the Kola Peninsula. Therefore, milk (reindeer, goat and cow) made no significant contribution to the nutrient intake before modern times among the Sami people, whereas the Kola Sami did not use reindeer milk as food. Cow's milk has been available for approximately 170–200 years (10).

## The aim of the study

The primary aim of this study was to compare the prevalence of self-reported stomach symptoms after consuming milk, to study the intake of dairy products, and to study self-reported stomach abdominal pain among Sami and non-Sami in Northern and Mid-Norway.

## Material and methods

### The SAMINOR study

The present study is based on data from the population-based study of health and living conditions in areas with mixed Sami, Kven and Norwegian majority populations (the SAMINOR study), for which data were collected for 2003 and 2004. Questions about self-reported milk intolerance, stomach problems, ethnicity, area of residence and intake of milk and dairy products were collected by means of 3 different questionnaires. The questionnaires were self-administered, but the respondents were reminded to fill out the questions about ethnicity during screening. The questionnaires were available both in the Norwegian and Sami languages. Further details on the collection process and methods have been published previously by Lund et al. (18).

### Geographical area

**Fig. 1:**
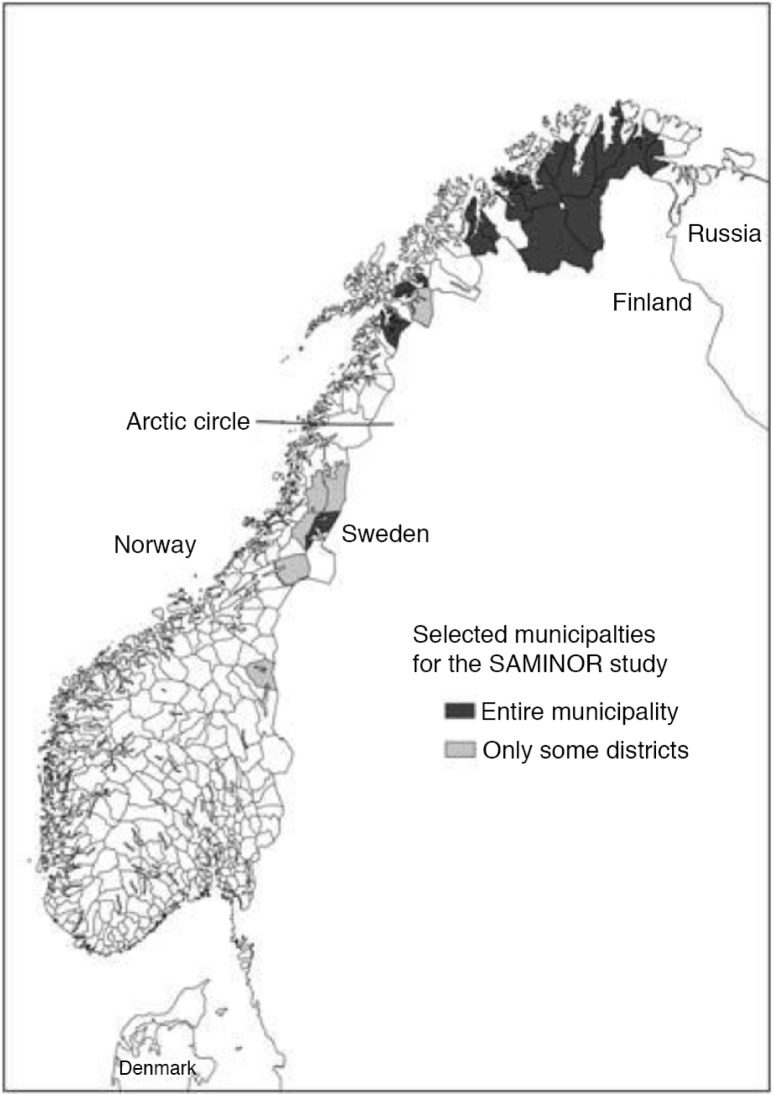
Municipalities investigated in the SAMINOR study. Finnmark county: Karasjok, Kautokeino, Porsanger, Tana, Nesseby, Lebesby, Alta, Loppa and Kvalsund. Troms county: Kåfjord, Kvænangen, Storfjord, Lyngen, Skånland and Lavangen. Nordland county: Tysfjord, Evenes and parts of Hattfjelldal, Grane and Narvik. Nord-Trøndelag county: Røyrvik and parts of Namskogan and Snåsa. Sør-Trøndelag county: part of Røros.

The study intended to cover all municipalities in Norway where more than 5–10% of the population reported themselves as Sami in the 1970 census (19), based on the definition of Sami as a person with at least one grandparent who spoke the Sami language at home. In addition, some selected districts were included from municipalities with an overall lower proportion of subjects with Sami ethnicity. Altogether, 24 municipalities, often referred to as the core area for Sami settlement, stretching from Mid-Norway to the Russian border in Northern Norway, were included in the survey (18). Figure 1 shows the geographical areas referred to as the SAMINOR area in the text.

### Sample

People aged between 36 and 79 living in the SAMINOR area (Fig. 1) (a total of 27,151 persons) were invited to participate in the SAMINOR study, of which 16,538 chose to participate and gave informed consent to medical research, giving a response rate of 61%. Included in the analysis were respondents who reported self-reported stomach symptoms/abdominal pain and ethnicity (n=15,546). The response rate was highest in areas where Sami was the majority. The ethnic distribution was found to be Sami (33.4%), Kven (7.3%) and Norwegian majority population (57.2%).

We have little information about non-respondents other than that they tend to be young, single males. With a participation rate of 61%, selection bias is a possibility (18). The differences between respondents and non-respondents are often important but rarely significant enough to undermine studies (20).

### Ethnicity

The questionnaire asked about the language used at home by the participants, their parents and grandparents, with the available choices being *Sami, Norwegian, Kven or Other* (to be specified). Questions regarding the ethnic background of participants and their parents were linked with the same 4 response options. In addition, participants were asked about self-perceived ethnicity. Participants were allowed to provide more than one response to every question contained in the questionnaire. With regard to responses to questions about ethnicity, 3 categories were developed:

- Sami: Respondents reported Sami language or ethnicity.
- Kven: Descendants of Finnish-speaking immigrants from northern Finland and Sweden.
- Norwegian majority population: Participants reporting no Sami, Kven or foreign affiliation.

In this study, Kvens are defined as participants reporting that Kven language or ethnicity applies to themselves, one of their parents or one of their grandparents. Since we are particularly interested in the Sami population, participants with dual Sami and Kven backgrounds are defined as Sami.

In Norway, no systematic registration of ethnicity is available for research purposes, except for the last census in 1970. To capture ethnicity (Sami, Kven or majority Norwegians), the SAMINOR study included questions on the language used at home by the grandparents, parents and the subject, in addition to the ethnic background of the parents and the subjects, and also a question on self-perceived ethnicity. Based on these questions, ethnicity was divided into 3 groups: Sami, Kven and majority Norwegians.

### Self-reported stomach symptoms/abdominal pain

Self-reported stomach symptoms after consuming milk were measured using the following question: “Have you had stomach/intestinal symptoms after consuming milk?” Available responses were “Yes” or “No”. Information was, however not collected on time period between consumption and pain.

The question on stomach pain was as follows: “Have you ever had pains/aches in your stomach lasting for at least two weeks?” Available responses were “Yes” or “No”, and “If yes, where in the stomach are the pains located? (mark only one). Available responses were “Upper part”, “Lower part” or “Entire stomach.” Then followed a question about the duration: “Normally, for how long are the stomach pains present?” Available responses: “For periods of weeks,” “For periods of months” and “Always.”

Other self-reported stomach symptoms were measured using the following question: “Do you often suffer from flatulence, rumbling in the stomach or much wind?” Available responses were “Yes” or “No.”

Stomach symptoms in other family members were measured using the question: “Are there others in your family with similar stomach symptoms?” Available responses were “Mother,” “Father,” “Siblings,” “Children” or “Nobody.” During our analysis, the variable was dichotomized into “yes” or “no.”

### Intake of dairy products

Intake of milk products was measured by the following 4 questions: “How much do you normally drink of the following? ‘Full milk, full-fat cultured milk and yoghurt’, ‘Semi-skimmed milk, semi-skimmed cultured milk and low-fat yoghurt’, ‘Skimmed milk and skimmed cultured milk’ and ‘Extra semi-skimmed milk’.” Available responses were: “Rarely/Never,” “1–6 glasses per week,” “1 glass per day,” “2–3 glasses per day” or “4 glasses a day or more.” During analysis, the responses into: “>2 glasses per day,” “1 glass per day,” “Weekly” or “Rarely/Never.”

Intake of cheese was measured by: “How often do you usually eat cheese (all types)?” Available responses were: “Rarely/Never,” “1–3 times per month,” “1–3 times per week,” “4–6 times per week,” “1–2 times per day” or “3 times or more per day.” During analysis we categorized it into: “Every day,” “Weekly,” “Monthly” or “Rarely/Never.”

### Other variables

The respondents’ level of education was categorized according to how many years they had spent in educational institutions (including primary and secondary school), with the response options “Low” (less than 10 years), “Medium” (between 10 and 13 years) and “High” (more than 13 years).

### Ethics

Ethical approval was granted by the Regional Committee for Medical and Health Research Ethics in Northern Norway and the Norwegian Data Protection Authority.

### Data analysis

IBM SPSS Statistics software for MAC version 22 and STATA version 13.1 (Stata Corp, College Station, TX) were used for data processing and statistical analysis. For categorical variables, we used Pearson's chi-square tests to assess the differences in distribution between groups. In Table III, one-way ANOVA test between the ethnic groups was used. In Table IV, Jonckheere Trend Test was used. The age-adjusted prevalence rates presented in Table V were based on logistic regression estimates. The results are presented as odds ratios (OR) to indicate risk with a 95% confidence interval (CI). Logistic regression was performed to evaluate the changes in the effect of the main exposure on self-reported stomach symptoms after consuming milk products. We adjusted the analyses for age (as a continuous variable), intake of milk and cheese products and general abdominal pain.

## Results

Selected characteristics of the study are presented in Table I. The mean age of the sample was 54.4 years. One third of the study sample reported Sami affiliation. Fifty two per cent were females and 79% of the sample lived on the Norwegian coast.

**Table I:** Characteristics of study sample (15,546)^a^

Characteristic	*n*	%
Age (years)		
36–49	5,663	36.4
50–64	6,573	42.3
65–79	3,310	21.3
Gender		
Males	7,461	48.0
Females	8,085	52.0
Ethnicity		
Sami	5,199	33.4
Kven	1,140	7.3
Majority Norwegians	8,898	57.2
Geographical area of residence		
Inland	3,259	21.0
Coast	12,287	79.0
Level of education		
Low	5,432	38.0
Medium	5,176	36.2
High	3,695	25.8

a n may not total to 15,546 for all variables due to missing values.

The prevalence of self-reported stomach symptoms after consuming milk was found to be 27.1% among Sami females and 17.6% among Sami males compared to 17.3 and 10.7% among majority Norwegian peers, respectively (Table II). The prevalence of self-reported stomach symptoms after consuming milk among Sami with 4 Sami-speaking grandparents was the same as in Sami reporting any kind of Sami origin (data not shown). The Kven population also reported more symptoms after consuming milk. There were also ethnic differences in reporting general abdominal pain, with Sami females reporting the highest prevalence (24.7%). Sami reported more abdominal pain in the whole stomach (29.8%). On the question regarding flatulence, rumbling in the stomach or much wind, Sami (44.6%) and Kven (46.2%) females reported the greatest prevalence. Sami (females 48.8%, males 29.8%) and Kven (females 49.8%, males 33.1%) reported higher for family members with stomach symptoms than the other Norwegians (females 40.1%, males 22.9%). In total, the Sami and Kven had more stomach pain/symptoms, including self-reported stomach symptoms after consuming milk, than the majority Norwegians.

**Table II:** Prevalence rates* of self-reported symptoms after consuming milk and stomach pain/problems by ethnic groups: the SAMINOR study

	Sami	Kvens	Majority Norwegians	Total	
	% (*n*)	% (*n*)	% (*n*)	% (*n*)	*p* †
Self-reported symptoms after milk consumption^a^					
Males					
Yes	17.6 (425)	14.4 (77)	10.7 (425)	13.5 (927)	
No	82.4 (1,980)	85.2 (460)	89.3 (3,514)	86.5 (3,514)	<0.001
Females					
Yes	27.1 (654)	19.9 (103)	17.3 (758)	17.4 (758)	
No	72.9 (1,750)	80.1 (422)	82.7 (3,586)	82.6 (3,586)	<0.001
Self-reported stomach pain‡^b^					
Males					
Yes	20.7 (522)	22.5 (127)	17.3 (760)	19.8 (1,409)	
No	79.3 (1,983)	77.5 (432)	82.7 (3,309)	80.2 (5,724)	0.02
Females					
Yes	24.7 (616)	25.0 (137)	20.3 (920)	22.1 (1,673)	
No	75.3 (1,881)	75.0 (407)	79.7 (3,612)	79.7 (3,612)	<0.001
Location of pain					0.14§
Upper part	51.0 (580)	54.3 (143)	53.8 (904)	53.0 (1,627)	
Lower part	19.2 (219)	21.9 (58)	20.1 (338)	20.4 (615)	
Entire stomach	29.8 (339)	23.7 (63)	26.1 (438)	26.5 (840)	
Duration of pain					0.35§
Weeks	73.4 (835)	72.0 (190)	70.6 (1,186)	72.0 (2,211)	
Months	15.5 (177)	18.6 (49)	16.9 (284)	17.0 (510)	
Always	11.1 (126)	9.3 (25)	12.5 (210)	10.9 (361)	
Self-reported stomach symptoms^c^					
Males					
Yes	35.3 (834)	33.8 (180)	33.4 (1,299)	34.3 (2,313)	
No	64.7 (1,528)	66.1 (351)	66.2 (2,543)	66.2 (2,543)	0.47
Females					
Yes	44.6 (1,056)	46.2 (237)	39.9 (1,688)	42.0 (2,981)	
No	55.3 (1,309)	53.6 (274)	60.1 (2,541)	58.0 (4,124)	<0.001
Other family members with stomach symptoms^d^					
Males					
Yes	29.8 (394)	33.1 (101)	22.9 (489)	26.3 (984)	
No	70.2 (920)	66.9 (207)	77.1 (1,625)	76.9 (1,625)	<0.001
Females					
Yes	48.8 (683)	49.5 (142)	40.1 (963)	43.8 (1,788)	
No	51.2 (711)	50.5 (151)	59.9 (1,434)	56.2 (2,296)	<0.001
Total stomach pain/symptoms, including symptoms after milk consumption^e^					
Males					
Yes	48.3 (1,067)	47.8 (237)	44.3 (1,608)	46.0 (2,912)	
No	51.7 (1,140)	52.2 (259)	44.3 (1,608)	54.0 (3,417)	0.009
Females					
Yes	57.3 (1,246)	55.9 (264)	49.6 (1,944)	52.6 (3,454)	
No	42.7 (930)	44.1 (209)	50.4 (1,974)	47.4 (3,113)	<0.001

* The age-adjusted probabilities are based on logistic regression estimates.

† p Value from chi-square test for difference between ethnicity groups.

‡ Self-reported stomach symptoms were measured using the following question: “Do you often suffer from flatulence, rumbling in the stomach or much wind?”

§ p Value from chi-square test for difference between either location of pain or duration of pain and ethnicity.

a The numbers of non-responders for the variable were 7.8% (n=580) for males and 10.0% (n=812) for females.

b The numbers of non-responders for the variable were 4.4% (n=328) for males and 6.3% (n=512) for females.

c The numbers of non-responders for the variable were 9.7.4% (n=726) for males and 12.1% (n=980) for females.

d The numbers of non-responders for the variable were 49.9% (n=3,725) for males and 49.5% (n=401) for females.

e The numbers of non-responders for the variable were 15.2% (n=1,132) for males and 18.8% (n=1,518) for females.

Table III shows data on the frequency of consumption of dairy products. Milk consumption was high among all the ethnic groups (Sami, Kven and majority Norwegians); however, there are some ethnic differences, where more Sami never (or rarely) consume milk. Similar patterns were found for the consumption of cheese.

**Table III:** Intake of milk products by gender and ethnic groups: the SAMINOR study

	Sami		Kvens		Majority Norwegians		*p* a
		
	%	*n*	%	*n*	%	*n*	
Milk products							
Males							0.005
>2 glasses per day	39.4	992	40.6	232	41.5	1,705	
1 glass per day	21.1	531	22.2	127	23.8	978	
Weekly	25.2	634	24.5	140	22.8	934	
Rarely/Never	14.4	363	12.6	72	11.9	487	
Females							<0.001
>2 glasses per day	23.1	586	20.8	115	19.0	875	
1 glass per day	26.6	674	28.0	155	29.3	1,349	
Weekly	28.8	730	30.3	168	32.0	1,472	
Rarely/Never	21.6	548	20.9	116	19.7	907	
Cheese							
Males							<0.001
Every day	30.4	743	35.7	198	38.9	1,565	
Weekly	51.9	1,270	50.4	279	50.6	2,033	
Monthly	10.5	257	8.1	45	6.8	275	
Rarely/Never	7.3	178	5.8	32	3.7	147	
Females							<0.001
Every day	48.5	1,210	51.9	280	51.6	2,342	
Weekly	41.9	1,047	41.9	226	42.6	1,931	
Monthly	5.7	143	3.0	16	3.8	173	
Rarely/Never	3.9	97	3.2	17	2.0	89	

a p Values from chi-square test for difference between ethnic groups.

For the variable milk products, the missing were 2.3% (n=362), and for cheese products, the missing were 4.3% (n=665).

There were no significant differences in intake of dairy products between ethnic groups among those with selfreported symptoms after consuming milk, except for the intake of cheese products among females, where more Sami females than non-Sami females never (or rarely) consume cheese (Table IV).

**Table IV:** Intake of milk products by self-reported symptoms after milk consumption, ethnicity and gender: the SAMINOR study

	Self-reported symptoms after consuming milk			*p* a
	
	Sami Yes % (*n*)	Kvens Yes % (*n*)	Majority Norwegians Yes % (*n*)	
Milk products				
Males				**0.37**
>2 glasses per day	14.7 (61)	14.3 (11)	13.0 (54)	
1 glass per day	12.0 (50)	15.6 (12)	17.8 (74)	
Weekly	28.9 (120)	32.5 (25)	28.0 (116)	
Rarely/Never	44.3 (184)	37.7 (29)	41.2 (171)	
Females				**0.65**
>2 glasses per day	8.4 (53)	7.9 (8)	6.7 (50)	
1 glass per day	20.4 (129)	16.8 (17)	18.8 (139)	
Weekly	28.3 (173)	32.7 (35)	32.5 (241)	
Rarely/Never	42.9 (271)	42.6 (43)	42.0 (311)	
Cheese				
Males				**0.62**
Every day	34.1 (140)	29.7 (22)	37.8 (157)	
Weekly	45.9 (187)	50.0 (37)	46.3 (192)	
Monthly	9.6 (39)	9.5 (7)	8.9 (37)	
Rarely/Never	10.1 (41)	10.8 (8)	7.0 (2.9)	
Females				**0.003**
Every day	47.7 (299)	46.9 (46)	46.6 (343)	
Weekly	35.9 (225)	43.9 (43)	42.8 (315)	
Monthly	8.1 (51)	1.0 (1)	6.0 (44)	
Rarely/Never	8.3 (52)	8.2 (8)	4.6 (34)	

a p Values from chi-square test for difference between ethnic groups.

For the variable milk products, the missing were 2.3% (n=362), and for cheese products, the missing were 4.3% (n=665).

OR for self-reported stomach symptoms after consuming milk by gender and ethnicity are presented in Table V. The age-adjusted models show a significant effect of Sami ethnicity in both men and woman on self-reported stomach symptoms after consuming milk. In males, the OR was 1.78 (p=0.001) and in females 1.77 (p=0.001). A significant effect (1.40) was also observed in Kven males (p=0.03).

**Table V:** Odds ratio estimates of self-reported stomach symptoms after consuming milk products by ethnic groups: the SAMINOR study

	Age-adjusted				Full model^a^			
	
	Males		Females		Males		Females	
			
	OR	95% CI	OR	95% CI	OR	95% CI	OR	95% CI
Sami	1.78	1.54–2.06	1.77	1.57–1.99	1.69	1.43–1.99	1.79	1.57–2.05
Kven	1.40	1.08–1.82	1.18	0.94–1.49	1.34	1.01–1.80	1.13	0.88–1.46
Majority Norwegians	1		1		1		1	

a The full model was adjusted for: age (as a continuous variable), intake of milk and cheese products and general abdominal pain.

We also present a full (mutually adjusted) model. The full model produced OR of 1.77 (p=0.001) and 1.64 (p=0.001) for Sami females and males, respectively. Backward regression shows that age, level of education, abdominal pain and intake of dairy products had confounding or intermediate effects.

## Discussion

Sami respondents had a higher prevalence of self-reported stomach symptoms after consuming milk than the Norwegian majority population. The reporting was highest among Sami females. Kven people also reported more symptoms after consuming milk than the Norwegian majority population. There were also ethnic differences in reporting general abdominal pains, where Sami females reported the highest prevalence. Sami reported more stomach pain in the entire stomach. Sami and Kven reported higher for family members with stomach symptoms than the other Norwegians.

Consumption of milk and dairy products (yoghurt and cheese) was high among all the ethnic groups. However, significantly more Sami than non-Sami never (or rarely) consume milk or cheese and individuals who reported stomach symptoms after consuming milk had an significant lower intake of dairy products than those not reporting stomach symptoms after consuming dairy products. In total, the Sami and Kven had more general stomach pain/symptoms, including self-reported stomach symptoms after consuming milk than the majority Norwegians. These findings indicate a higher prevalence of milk intolerance in the Sami and Kven populations than in the Norwegian majority population. These finding suggest that health professionals should be made aware that some of the reported stomach problems Sami experience could possibly be related to intake of dairy products.

The indigenous circumpolar populations have previously been studied to investigate the prevalence of lactose intolerance (13, 21). Findings have shown the frequency of lactose intolerance to be relatively higher in indigenous groups in the North than in their majority peers (10). The ability to drink milk as an adult occurs at a high frequency among the majority of Northern Europeans that have practised dairying and cattle rearing. It has been suggested that this correlation represents a case of gene-cultural co-evolution, that is, an adaptive genetic trait exposed to positive selection induced by cultural practices (9). Hence, the development that led to the modern Northern European lifestyle, which is strongly based on dairy and other farm products, may have been a process where cultural practices and genes interacted (9). In areas where dairying and adult milk consumption had a short tradition, the prevalence of lactose intolerance is high. On the contrary, most of the European populations, where dairy farming has had a long tradition, reported a lower prevalence of lactose intolerance (10). The higher prevalence we found among Sami of self-reported stomach symptoms after consuming milk is in accordance with their short tradition of dairying and adult milk consumption. The literature has shown that milk (reindeer, goat and cow) made no significant contribution to the nutrient intake before modern time among the Sami people (12), and cow's milk has only been available for about 170–200 years among the Sami people in the High North (10).

Previous studies showing that subjects with intolerance to lactose tend to reduce their consumption of milk and dairy products (8). Individuals with self-reported stomach symptoms after consuming milk had a significantly lower intake of milk products than subjects without self-reported stomach symptoms after consuming milk. Thus, self-reported stomach symptoms after consuming milk had a significant effect on the milk consumption of the individuals in our study. The presence of self-reported stomach symptoms after consuming milk resulted in a reduction in the daily intake of dairy products.

### Strengths and weaknesses

The relatively high participation rate in our study indicates that the findings of this study could be representative of the Sami population living in Norway. The study contributes empirical evidence of the prevalence of self-reported stomach symptoms after consuming milk and general stomach abdominal pain among the Sami population. However, some limitations need to be noted, such as the fact that the validity of our questions about self-reported stomach symptoms after consuming milk, self-reported abdominal pain and other self-reported stomach problems has to our knowledge not been assessed with regard to relevant gold standards for the diagnosis of primary lactose intolerance. A part of those reporting abdominal symptoms after milk intake might not be due to intolerance to milk but rather due to irritable bowel syndrome. Self-reported milk intolerance shows sensitivities from 30 to 70% and specificities from 25 to 87% (22). Self-reported stomach symptoms after consuming milk are not synonymous with primary lactose intolerance. Carroccio et al. (23) showed in their study that in individuals with self-reported milk intolerance, only 5% had lactase deficiency and lactose intolerance simultaneously, while 50% had lactase deficiency but tolerated milk, while about 40% in their study were lactose persistent and tolerated milk. Further, in the total population, 36% had lactase deficiency, while only 11% of these had clinical symptoms.

We have little information about non-respondents, other than that they tend to be young, single and males. With a participation rate of 61%, and minor missing values on stomach symptoms after consuming milk [7.8% (n=580) for men and 10.0% (n=812) for females], selection bias is a possibility. However, the differences between respondents and non-respondents are often important but rarely significant enough to undermine studies (20).

## Conclusions

Our study has shown that the Sami population reported more stomach symptoms after consuming milk, suggesting a higher prevalence of milk intolerance among the Sami population compared to the Norwegian majority population.

Further studies are necessary to determine the extent of primary lactose intolerance among Norwegian Sami and the population in Northern Norway in general.
